# Zap-X Radiosurgery System: Initial Clinical Experience in a Recurrent Gamma Knife Case Series

**DOI:** 10.7759/cureus.74005

**Published:** 2024-11-19

**Authors:** Ayako Horiba, Motohiro Hayashi, Takashi Maruyama, Ryutaro Nomura, Phyo Kim, Takakazu Kawamata

**Affiliations:** 1 Department of Neurosurgery, Tokyo Women’s Medical University, Tokyo, JPN; 2 Department of Neurosurgery, Utsunomiya Brain and Spine Center, Utsunomiya, JPN; 3 Department of Neurosurgery, Kamiyacho Neurosurgical Clinic, Tokyo, JPN; 4 Department of Neurosurgery, Tokyo Women's Medical University, Tokyo, JPN

**Keywords:** gamma knife, neuro oncology, recurrence, stereotactic radiosurgery srs, zap-x

## Abstract

Introduction: The ZAP-X® Gyroscopic Radiosurgery System (ZAP Surgical Systems, Inc., San Carlos, CA, USA) is expected to be a highly accurate next-generation treatment system that enables gyro-stereotactic irradiation of intracranial lesions. In this study, we report the initial treatment course using ZAP-X for intracranial lesions that recurred after Gamma Knife (GK) treatment.

Methods: Patients with intracranial tumors treated with ZAP-X from April to August 2023 who relapsed after GK therapy were eligible for participating in this study. The primary endpoint was the progression control rate at six months; the secondary endpoints were the rate of target lesion reduction, treatment effect by tumor doubling time (TDT) before ZAP-X intervention, and adverse events.

Results: Six lesions were evaluated in six patients (two meningiomas, one solitary fibrous tumor, one auditory neuroma, one glomus tumor, and one metastatic tumor). The mean follow-up period was 10.6 months after treatment. The mean lesion volume was 5.3 cm^3^; the mean TDT was 241 days. The six-month progression control rate was 100%, with two partial responses and four stable diseases at the last evaluation. Adverse events were limited to only imaging changes in one patient (16.6%), indicating a significant correlation between ZAP-X pre-intervention TDT and post-intervention reduction rate.

Conclusions: The initial results of ZAP-X treatment in patients with recurrent disease following GK are reported for the first time. ZAP-X demonstrated potent anti-tumor efficacy and adequate tolerability and should be considered a new treatment option for relapsed refractory patients. Future studies should increase the number of cases and follow-up periods to further evaluate treatment outcomes and adverse events.

## Introduction

Stereotactic radiosurgery (SRS), including Gamma Knife (GK) surgery, is a versatile and well-established treatment of various intracranial tumors. However, when recurrence occurs after radiotherapy for difficult-to-remove or residual lesions, treatment options are very limited. Several linear accelerator (LINAC)-based platforms are currently used for SRS treatment. The recently developed ZAP-X system (ZAP Surgical Systems, Inc., San Carlos, CA, USA), with a maximum energy of 3 MV and source-to-axis distance (SAD) of 45 cm, allows steep dose gradients [1]. This technological advancement could improve the availability of SRS and potentially address recurrent cases after GK treatment [2]. There are only a few reports and treatment results on the use of ZAP-X in clinical practice. This study aimed to report the initial clinical course using ZAP-X treatment in patients with recurrence after GK treatment.

## Materials and methods

The study included six patients with six lesions who relapsed after GK treatment and were treated with ZAP-X between April and August 2023. All patients had previously undergone surgery and had a confirmed pathological diagnosis. The diagnosis of recurrence after GK therapy was defined as a more than 20% enlargement in lesion size on magnetic resonance imaging and is based on cases in which imaging evaluation by MRI was available for at least six months after ZAP-X treatment. Tumor volume was measured by manually drawing the tumor contour on the treatment planning device.

The ZAP-X comprises of a compact 3 MV LINAC, eight different collimator wheels ranging between 4 and 25 mm, and a kV image guidance system. Treatment planning was performed using either "forward-planning" or "forward + inverse-planning". In "forward-planning," isocenters were manually placed in the target by the planner. In "forward + inverse-planning," isocenters were manually placed first, followed by inverse optimization of beam weights and manual addition, deletion, or repositioning of isocenters. These were repeated several times.

The follow-up parameter was local tumor recurrence, defined as an increase in tumor volume. Patients were followed regularly every three months after radiotherapy, with monthly follow-ups thereafter if clinically required. Data collection included pretreatment clinical history, pathological diagnosis, radiographical features, treatment parameters, and clinical and radiographical follow-up. To assess the growth rate of each tumor, the tumor doubling time (TDT) of tumor volume was calculated. Assuming that changes in tumor volume are exponential, a regression line, log y = log a + bx (x: years after the baseline radiologic image, y: tumor volume), was calculated by nonlinear square regression. The TDT of tumor volume was defined as (log2)/b. TDT was calculated from the baseline and historical images performed before. 

The relationship between tumor doubling time (TDT) before treatment and post-treatment shrinkage rate was examined using bivariate analysis (t-test). Data are expressed as mean ± standard deviation. JMP Pro Version 17 (SAS Institute, Cary, NC, USA) was used for statistical treatment, and p<0.05 was considered significant in the analysis.

## Results

Six patients with a total of six lesions (including two meningiomas, one solitary fibrous tumor, one acoustic schwannoma, one paraganglioma, and one metastatic brain tumor) were included. The mean follow-up period after treatment was 10.6 months (standard deviation: 1.04 months). The median age of the patients at the time of treatment was 59.8 years, with five out of six being male patients.

**Figure 1:**
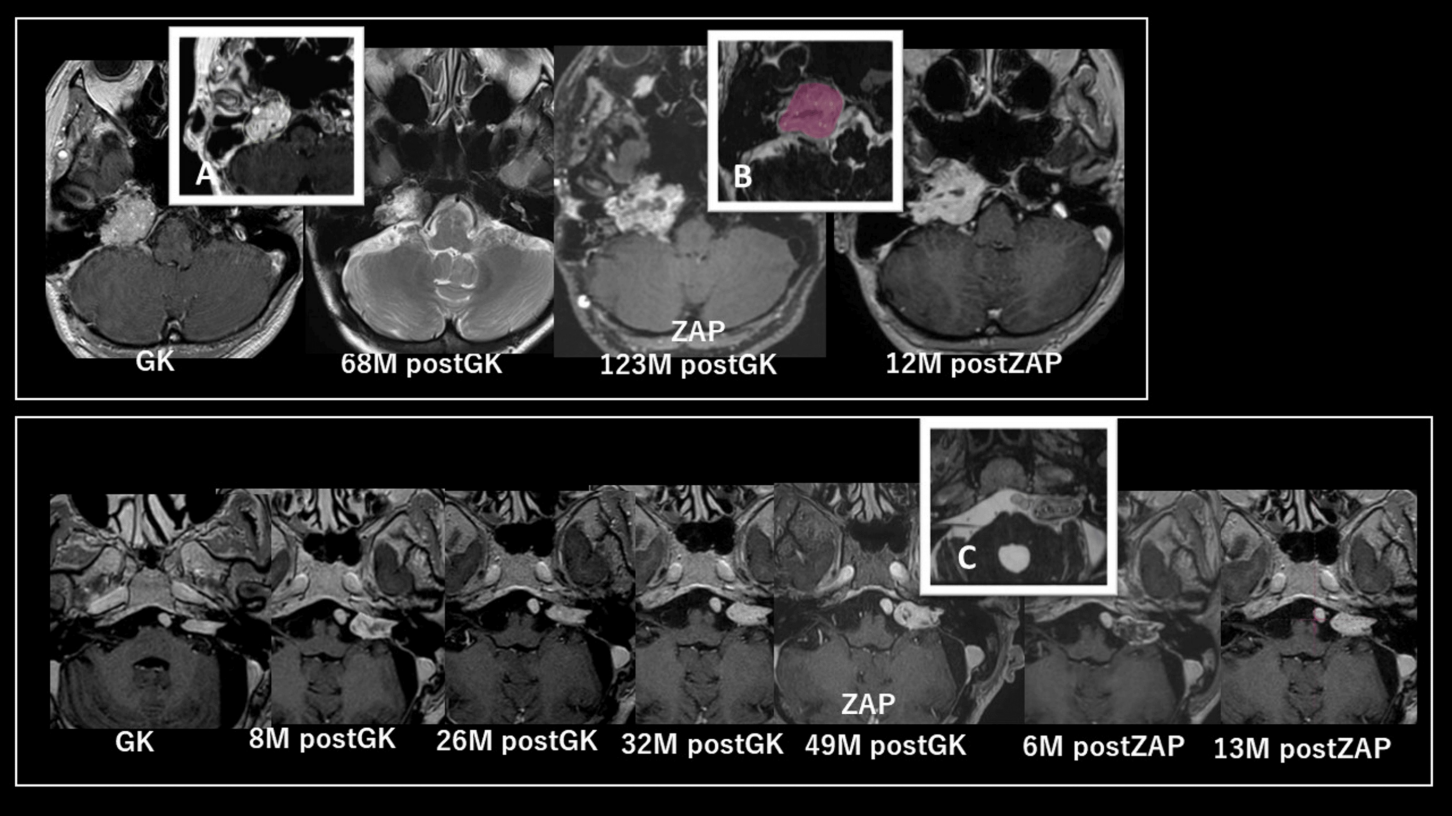
Time course of case 2 (upper row: paraganglioma) and case 3 (lower row: acoustic schwannoma). Image (A) shows the Gamma Knife treatment dose, with the yellow line representing the treatment dose range. Images B and C show ZAP-X treatment doses, with red indicating the target, yellow and green indicating the treatment dose range, and blue indicating the low dose range. GK: gamma knife, Zap: Zap-X.

**Figure 2:**
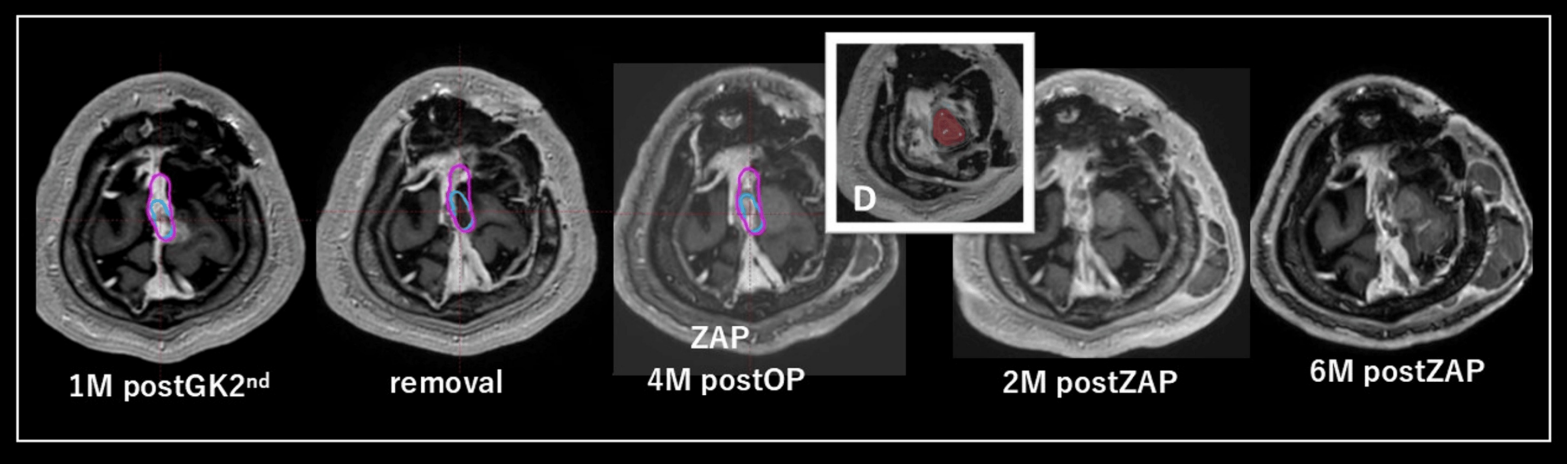
Time course of case 6 (anaplastic meningioma). Image (D) shows the treatment dose of ZAP-X. The red line is the treatment dose range, and the blue line is the low dose range. GK: gamma knife, Zap: Zap-X.

**Figure 3:**
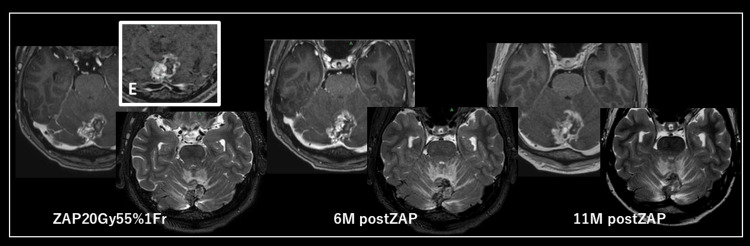
Time course of case 4 (lung metastatic brain tumor). Image (E) shows the ZAP-X treatment dose; the peach line shows the treatment dose range, and the blue line shows the low dose range.

**Figure 4:**
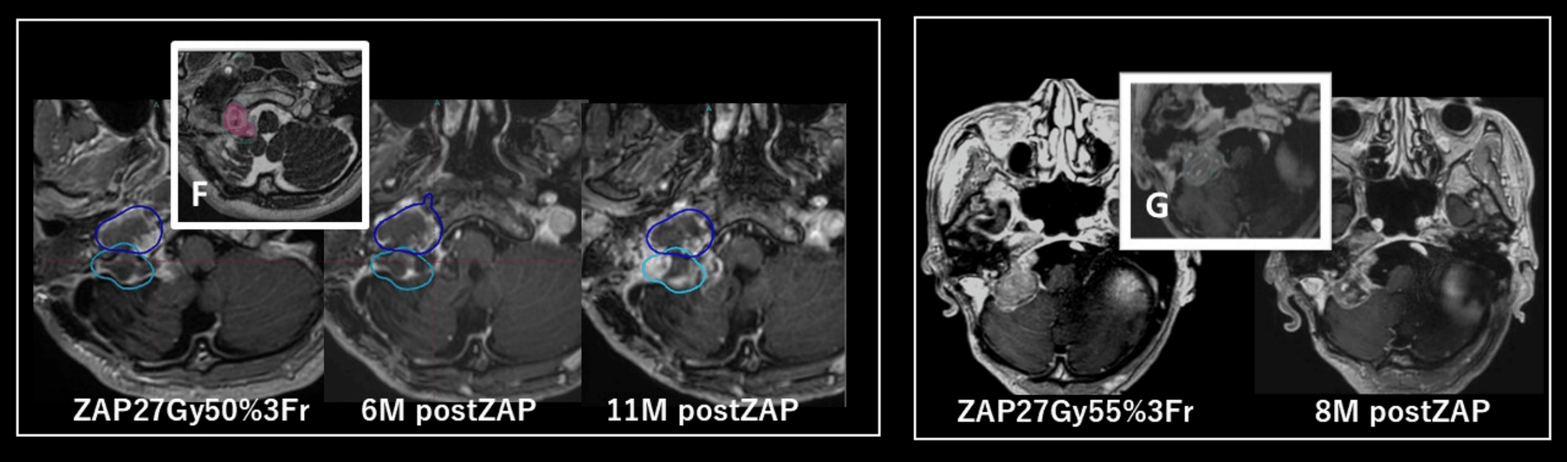
Time course of case 1 (left: solitary fibrous tumor) and case 5 (right: meningothelial meningioma). Images (F) and (G) show the ZAP-X treatment dose. Blue and light blue lines indicate previous gamma knife treatment areas. The green line indicates the treatment dose range, and the blue line indicates the low dose range. The red line shows the target area, and the orange line shows the high-dose area.

The mean time from GK diagnosis of relapse to treatment was 17 months (standard deviation: 14.2 months). The mean lesion volume was 5.3 cm^3^ (standard deviation: 4.5 cm^3^); the mean TDT was 241 days (standard deviation: 273 days). The six-month progression control rate was 100%, with lesion reduction ranging between −11% and 48% (mean: 16.7%). At the last evaluation, two patients showed partial response (PR), and four patients showed stable disease. Adverse events were observed in only one patient (16.6%), limited to imaging changes. A correlation was found between ZAP-X® pre-intervention TDT and post-intervention reduction rate (p = 0.038). Detailed patient data are summarized in Table 1; treatment parameters are listed in Table 2. The progress of six cases is shown in the images, but three cases are highlighted in Figures 1-4.

**Table 1:** Patient data and clinical course summary. Dx: diagnosis, Tx: treatment, rec: recurrence, TV: tumor volume, Dia: diameter, TDT: tumor doubling time, FA: fraction, FU: follow up, SD: stable disease, PR: partial response, GKSRS: gamma knife stereotactic radiosurgery, GKSRT: gamma knife stereotactic radiotherapy.

Case	Sex	Age at ZAP-X	Final Dx	MIB-1 (%)	Prior Tx	Time to rec (M)	TV (Cm^3^)	Dia. (mm)	Tx dose (Gy)	TDT (day)	No. of FA Fx	Shrinkage rate (%)	FU (M)	Effect
1	M	56	Solitary fibrous tumor	NC	GKSRS	4	4.04	19.75	27	101	3	9.3	13	SD
2	F	39	Paraganglioma	NC	Embolus	17	14.9	30.5	16	303	1	3.9	12	SD
3	M	66	Acoustic schwannoma	2	GKSRS	46	2.85	17.58	25.5	756	3	-11.1	12	SD
4	M	48	Lung meta	－	GKSRT	17	1.62	14.59	20	68	1	30	11	PR
5	M	73	Meningothelial meningioma	1	GKSRS	16	5.96	22.49	20	213	1	20.4	11	SD
6	M	44	Anaplastic meningioma	40	Removal	3	2.74	17.35	30	8	3	48	6	PR
Mean±SD						17±14.2	5.3±4.5	20.4±5.1		241±243		16.7±18.9	10.8±2.3	

**Table 2:** Summary of treatment data. BED: biologically effective dose.

	Median	Mean	Range
Benign (four lesions)			
Planning target volume (cc)	5	6.9	2.8-14.9
Prescription dose (Gy)	26.25	23.8	16-27
Number of fractions	3	3	1 or 3
BED (α/β: benign 4)	83.9	83.8	79.7-87.8
Prescription isodose line (%)	50	51.2	50-55
Coverage (%)	90.2	87.8	74.9-95.8
New conformity index	1.24	1.36	1.06-1.41
Gradient index	3.02	3.03	2.98-3.06
Malignant (two lesions)			
Planning target volume (cc)	-	2.18	1.62-2.74
Prescription dose (Gy)	-	25	20-30
Number of fractions	-	2	1 or 3
BED (α/β: malignant 10)	-	60	60
Prescription isodose line (%)	-	52.5	50-55
Coverage (%)	-	87.3	81.4-93.1
New conformity index	-	1.26	1.25-1.27
Gradient index	-	2.5	2.47-2.53

Case 2

A 38-year-old woman was diagnosed with paraganglioma and initially treated with two embolization procedures and GK treatment following excision with high-flow bypass. Although the tumor size was reduced after treatment, it recurred 10 years later. Although the patient underwent a third embolization, the tumor re-enlarged. Subsequently, the patient was treated with SRS with ZAP-X. Since then, there has been consistent tumor shrinkage without symptoms (Figure 1, upper).

Case 3

At 62 years old, a patient underwent craniotomy for an acoustic schwannoma. Three years later, the residual tumor had enlarged and was treated with GK. After approximately two years, transient swelling due to inflammation subsided; the tumor regressed. However, six months later, the swelling reappeared. After the initial operation, the patient experienced facial paralysis, taste disturbance, and numbness and was highly resistant to another craniotomy. Therefore, ZAP-X treatment was performed approximately four years after GK. After the procedure, transient swelling appeared earlier than that with GK; signs of response convergence without worsening symptoms were observed approximately one year after ZAP-X. This is the only case in this study wherein tumor shrinkage was not achieved immediately after treatment. However, it is likely to be a pseudoprogression observed after SRS treatment, including GK; further tumor shrinkage and a reduction in inflammation are expected in the future (Figure 1, lower).

Case 6

The patient had an initial tumor removal at the age of 40, followed by four additional surgeries and three GK treatments due to repeated recurrences. The pathological diagnosis was anaplastic meningioma (MIB-1: 60%); the patient was treated with ZAP-X due to rapidly increasing recurrence three months after a total resection. Two months later, there was significant tumor shrinkage. Approximately seven months after ZAP-X treatment, the patient died from pulmonary metastasis from the meningioma (Figure 2).

## Discussion

We report our initial experience in ZAP-X treatment in six patients with intracranial tumors that recurred following GK treatment. The patient group in this study included those who experienced relapse after GK treatment, wherein the risk of radiation injury or acquisition of treatment resistance due to GK re-irradiation was a strong concern, convincing them to consider surgical resection as a treatment option before introducing ZAP-X.

After starting ZAP-X treatment, we observed a higher shrinkage effect after ZAP-X irradiation compared with GK. At the final evaluation, two patients had partial responses (PR) and four patients had stable disease. Imaging changes showed treatment-free cerebral edema (CTCAE ver5.0 grade 1) in one patient, and no adverse events of CTCAE ver5.0 grade 2 or higher were observed (Figure 3). We thought that ZAP-X might have a higher anti-tumor effect at the same dose and radiobiological effect (BED) as GK, making ZAP-X suitable for re-treatment. In addition, as expected, earlier tumor response and shorter time to achieve shrinkage were observed after ZAP-X compared with GK. There are only a few reports on treatment with ZAP-X. Although the observation period in this study was short, no adverse events were recorded. These findings suggest that ZAP-X is a tolerable treatment option as an initial experience [1-4]. TDT and shrinkage rate are well-known concepts in radiobiology, based on the law proposed by Bergonie and Tribondeau, which states that "undifferentiated, mitotic cells are radiosensitive." This principle suggests undifferentiation in the case of recurrent tumor cases [5]. Although differences between tumor tissues may have influenced the observed reduction times between treatment devices, we treated the same patient with GK and ZAP-X for recurrent dysplastic meningiomas at approximately the same time and observed a clear difference in response after both treatments. Here, we discuss the potential reasons for the faster reduction rate following ZAP-X treatment.

Characteristics of ZAP-X

The ZAP-X provides a steep dose distribution due to the low-energy treatment of 3 MV, which reduces scattered radiation and a source-to-axis distance (SAD) of 45 cm. In comparison, GK therapy has a similar energy level but a shorter SAD of 40 cm. Paddick et al. compared the conformity index (CI) and gradient index (GI) of ZAP-X with six other platforms for both benign and malignant tumors across six cases. They found that ZAP-X was superior to CyberKnife (CK) and linear accelerator platforms in GI; there was almost no difference between GK and ZAP-X [6]. Marianayagam et al. compared the dose distribution of SRS for essential tremors between GK, CK, and ZAP-X. They reported that ZAP-X had the lowest V12 Gy, which was associated with a reduced risk of radiation injury. We suggest that the steep dose distribution provided by ZAP-X could enable safe and (therapeutically) effective treatment planning [7].

Dose rate difference

The discussion regarding dose-rate effects has been ongoing for some time. However, no definitive conclusions have been reached [8-10]. The dose rate of ZAP-X was 15 Gy/min, providing a consistently stable supply, whereas the dose rate of GK, which uses Co-60, decreased continuously from the time of replacement. Niranjan et al. reported no change in tumor cell survival in vitro following Co-60 source attenuation using the GK. More recently, several studies have shown that the BED was higher at short beam-on times in a high oxygen partial pressure environment and that this effect was more pronounced at high doses compared with low doses [11,12]. Ganz noted that dose-rate effects have been well-demonstrated in vitro. However, in vivo, evaluation is challenging due to complex responses, such as vascular changes and immune function [13]. Nevertheless, the study emphasized the importance of the dose-rate concept for hypofractionated radiation therapy or SRT. 

Clinically, the impact of dose-rate effects was initially evaluated in terms of functional disability, primarily trigeminal neuralgia, with mixed results [14,15]. The hypothesis that Co-60 attenuation might affect BED has been questioned by several authors, who suggest that treatment time could be more relevant [16,17]. Tuleasca et al. reported that in arteriovenous malformation cases treated with GK, the critical factors were BED and beam-on time, not treatment time, and that dose rate was not significantly associated with obstruction [18]. The study also indicated that beam-on time was the sole factor associated with complications.

Several studies have described dose-rate effects, mainly for benign intracranial tumors, which are less complicated by systemic treatment. However, most of these studies refer to symptom improvement and tumor recurrence rates, not to the speed or rate of tumor shrinkage, which was the focus of our study [15,16,19-22]. It is possible that benign tumors are less likely to exhibit dose-rate effects due to their slow spontaneous tumor growth and post-treatment changes. Some studies suggest that dose rate effects and differences in BED are not associated with tumor control or symptomatic improvement and that dose rate effects in vivo should be more carefully studied [23]. Reports on worsening neuropathy with increasing dose rates strongly suggest that safety and efficacy may be achieved by prescribing specific dose rates, BEDs, and physical prescription doses.

Dose calculation algorithm differences

ZAP-X employs a conventional treatment planning system that assigns electron densities to tissues using CT for planning and patient-specific heterogeneity correction. In contrast, GK uses the TMR10 dose calculation algorithm, which assumes that the density of the entire tissue is equivalent to that of water. Nakazawa et al. compared the treatment planning in 29 patients with auditory nerve tumors. The study examined the TMR 10 and convolution methods using heterogeneity correction calculations. Their findings indicated that the absolute dose estimated for the target varied from 1% to 7% [24]. Similarly, Peters et al. reported differences of up to 10% in the treatment planning for 56 auditory nerve tumors [25]. It has been previously reported that the greatest differences were observed in lesions within 1 cm from the skull, near the sphenoid wing and sagittal sinus [26,27].

In addition to the TMR 10 method, Ramachandran et al. compared the beam-on time between planning using computed tomography (CT) and the convolution method using cone-beam CT. The work reported that the beam-on time using the convolution method with planning using CT was probably accurate, while dose calculation by the TMR10 was underestimated [28]. Despite showing no statistically significant difference, we observed a shortening in the timing of tumor shrinkage following ZAP-X treatment compared with the conventional GK treatment course. Being aware of the differences in treatment algorithms and how they translate to target and organs at risk dosimetry is crucial. Direct comparisons between ZAP-X and GK should be made with great caution, even if the prescribed dose and BED were the same.

Limitation

This study demonstrated the benefit of ZAP-X in patients with recurrence after GK treatment. However, it is limited by the short observation period after treatment. This does not necessarily mean that ZAP-X is a superior treatment to GK. Randomized controlled trials and case-control studies would help further clarify the usefulness of ZAP-X.

## Conclusions

To the best of our knowledge, our study is the first report on the initial results of ZAP-X treatment in patients with disease recurrence after GK treatment. Although a comparison with GK should be made cautiously due to differences in dose calculation algorithms and dose rates, the strong anti-tumor effect and adequate tolerability suggest that ZAP-X may be a viable option in patients with relapsed refractory disease. It is essential to continue increasing the number of cases and extend the follow-up period to further evaluate treatment outcomes and adverse events.
